# Disruption of cortical cell type composition and function underlies diabetes-associated cognitive decline

**DOI:** 10.1007/s00125-023-05935-2

**Published:** 2023-06-23

**Authors:** Karis Little, Aditi Singh, Angel Del Marco, María Llorián-Salvador, Maria Vargas-Soria, Mireia Turch-Anguera, Montse Solé, Noëlle Bakker, Sarah Scullion, Joan X. Comella, Ingeborg Klaassen, Rafael Simó, Monica Garcia-Alloza, Vijay K. Tiwari, Alan W. Stitt, on behalf of the RECOGNISED consortium

**Affiliations:** 1 The Wellcome‑Wolfson Institute for Experimental Medicine, School of Medicine, Dentistry & Biomedical Science, Queen’s University Belfast, Belfast, Northern Ireland UK; 2 Division of Physiology, School of Medicine, University of Cadiz, Cadiz, Spain; 3 Instituto de Investigacion e Innovacion en Ciencias Biomedicas de la Provincia de Cadiz (INIBICA), Cadiz, Spain; 4 Department of Medicine, Universitat Autònoma de Barcelona (UAB), Barcelona, Spain; 5 Diabetes and Metabolism Research Unit, Vall d’Hebron Institut de Recerca (VHIR), Vall d’Hebron University Hospital, Barcelona, Spain; 6 Cell Signaling and Apoptosis Group, Vall d’Hebron Institut de Recerca (VHIR), Barcelona, Spain; 7 Departament de Bioquímica i Biologia Molecular i Institut de Neurociències, Universitat Autònoma de Barcelona (UAB), Bellaterra, Spain; 8 Centro de Investigación en Red en Enfermedades Neurodegenerativas (CIBERNED - ISCII), Madrid, Spain; 9 Ocular Angiogenesis Group, Department of Ophthalmology, Amsterdam UMC location University of Amsterdam, Amsterdam, the Netherlands; 10 Centro de Investigación Biomédica en Red de Diabetes y Enfermedades Metabólicas Asociadas (CIBERDEM-ISCIII), Madrid, Spain; 11 Institute of Molecular Medicine, University of Southern Denmark, Odense C, Denmark; 12 Danish Institute for Advanced Study (DIAS), Odense M, Denmark; 13 Department of Clinical Genetics, Odense University Hospital, Odense C, Denmark

**Keywords:** Cognitive decline, Cortex, Diabetes, Metabolism, Neuroscience, Neurovascular unit

## Abstract

**Aims/hypothesis:**

Type 2 diabetes is associated with increased risk of cognitive decline although the pathogenic basis for this remains obscure. Deciphering diabetes-linked molecular mechanisms in cells of the cerebral cortex could uncover novel therapeutic targets.

**Methods:**

Single-cell transcriptomic sequencing (scRNA-seq) was conducted on the cerebral cortex in a mouse model of type 2 diabetes (*db/db* mice) and in non-diabetic control mice in order to identify gene expression changes in distinct cell subpopulations and alterations in cell type composition. Immunohistochemistry and metabolic assessment were used to validate the findings from scRNA-seq and to investigate whether these cell-specific dysfunctions impact the neurovascular unit (NVU). Furthermore, the behavioural and cognitive alterations related to these dysfunctions in *db/db* mice were assessed via Morris water maze and novel object discrimination tests. Finally, results were validated in post-mortem sections and protein isolates from individuals with type 2 diabetes.

**Results:**

Compared with non-diabetic control mice, the *db/db* mice demonstrated disrupted brain function as revealed by losses in episodic and spatial memory and this occurred concomitantly with dysfunctional NVU, neuronal circuitry and cerebral atrophy. scRNA-seq of *db*/*db* mouse cerebral cortex revealed cell population changes in neurons, glia and microglia linked to functional regulatory disruption including neuronal maturation and altered metabolism. These changes were validated through immunohistochemistry and protein expression analysis not just in the *db/db* mouse cerebral cortex but also in post-mortem sections and protein isolates from individuals with type 2 diabetes (74.3 ± 5.5 years) compared with non-diabetic control individuals (87.0 ± 8.5 years). Furthermore, metabolic and synaptic gene disruptions were evident in cortical NVU cell populations and associated with a decrease in vascular density.

**Conclusions/interpretation:**

Taken together, our data reveal disruption in the cellular and molecular architecture of the cerebral cortex induced by diabetes, which can explain, at least in part, the basis for progressive cognitive decline in individuals with type 2 diabetes.

**Data availability:**

The single-cell sequencing data that supports this study are available at GEO accession GSE217665 (https://www.ncbi.nlm.nih.gov/geo/query/acc.cgi?acc=GSE217665).

**Graphical Abstract:**

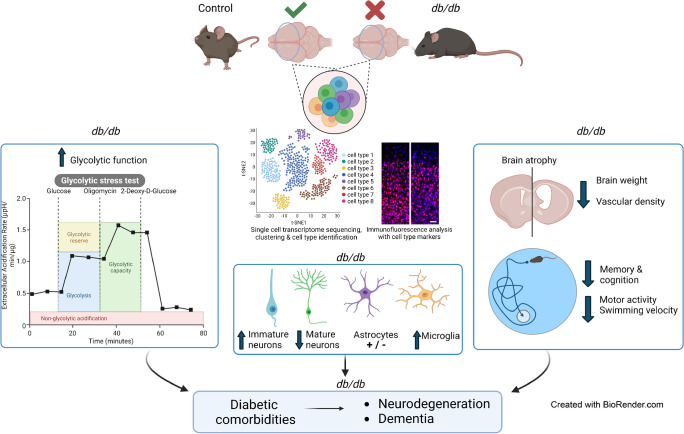

**Supplementary Information:**

The online version contains peer-reviewed but unedited supplementary material available at 10.1007/s00125-023-05935-2.


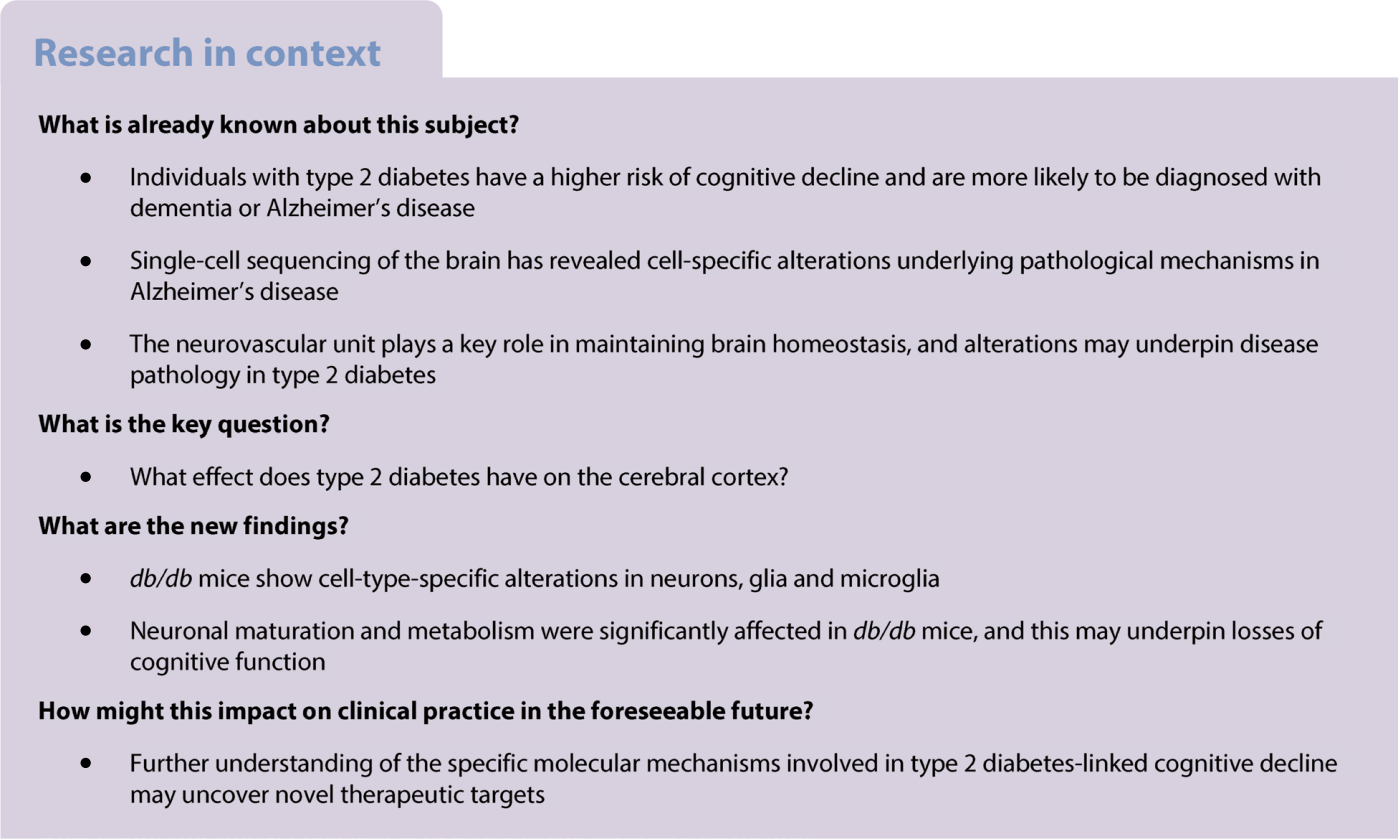


## Introduction

Type 2 diabetes constitutes a huge burden on healthcare systems worldwide due to multi-morbidities such as blindness, renal failure and cardiovascular-related death [1]. There is mounting evidence that type 2 diabetes is also associated with cognitive decline [2–4]. Individuals with type 2 diabetes have an approximate twofold increased risk of developing Alzheimer’s disease compared with non-diabetic, age-matched individuals [3].

Clinical evidence and pre-clinical data suggest that common pathways may be involved in type 2 diabetes and cognitive decline, including glial dysfunction, neurodegeneration and inflammation [5, 6], arising from dysfunctional insulin signalling, chronic hyperglycaemia and oxidative stress. These pathways may contribute to neurovascular unit (NVU) dysfunction in the brain and retina during type 2 diabetes [4]. However, the underlying pathways that drive neurodegeneration and cognitive decline in type 2 diabetes are not fully understood.

The NVU plays an important homeostatic role in the central nervous system and is formed by interdependency of vascular cells, glia, neurons and professional immune cells. Besides ensuring integrity of the blood–brain barrier, the NVU is important for immune privilege and regulating blood flow to meet the oxygenation and nutrient demands of the highly metabolically demanding neuropile. A recent study revealed human-specific transcriptomic signatures in the cerebrovasculature in Huntington’s disease [7] and another study revealed several Alzheimer’s disease risk genes mapping to endothelial and microglial cell populations [8]. Furthermore, neurodegenerative disease-linked shifts in brain cell populations have been reported [9]. Neuronal and glial population changes have been observed in Alzheimer’s disease [10] and may co-occur with reduced cortical thickness in individuals [11].

Analysis from a single-cell RNA sequencing (scRNA-seq) dataset revealed profound changes to the transcriptome of vascular, glial and neuronal cells from the retina of *db/db* mice, showing a useful linkage to the pathogenesis of diabetic retinopathy [12]. The scRNA-seq approach has also been used to characterise hippocampal changes in *db/db* mice, indicating the role of microglial populations in diabetes-induced cognitive decline [13]. The impact of type 2 diabetes on the brain NVU, particularly in the cortex, have not been fully explored. Moreover, whether the cognitive decline in type 2 diabetes is associated with disruption in brain cell populations remains unknown.

The aim of the present study was to provide novel insights into type 2 diabetes-associated cognitive decline and the underlying associated pathology in the cerebral cortex by combining state-of-the-art physiological and molecular measurements in *db/db* mice as a model and validation in human samples.

## Methods

### Animals

*db*/*db* (C57BLKS/heterozygous *db/+* mice [BKS.Cg-+*Lepr*^*db*^/+*Lepr*^*db*^/OlaHds]) (RRID: IMSR_ENV:HSD-173) were purchased from Harlan Laboratories (Netherlands) or Janvier (France). Since heterozygous (*db/+*) mice do not show a specific phenotype [14], wild-type (WT) and *db/+* mice are commonly considered a single control group [15, 16]. For For immunohistochemistry (IHC) and behavioural studies, WT mice (BKS.Cg-/OlaHds) belonging to the same colony were used as the control group. For transcriptional studies and cortical metabolic assessment, *db/+* mice were used as the control group. Mice were housed in a standard pathogen-free experimental facility, exposed to a 12 h light–dark cycle and had free access to food and water. Five or six male/female mice per group aged 14–16 weeks (representing an established but not yet advanced stage of diabetes) were used. Metabolic determinations (Table 1) were carried out as described in electronic supplementary material (ESM) Methods.

**Table 1:** Metabolic characterisation of 14-week-old *db*/*db* and control mice

Characteristic	Control mice	*db*/*db* mice
Body weight (g)	25.20 ± 2.17	41.66 ± 2.04**
Glucose (mmol/l)	6.92 ± 0.25	31.00 ± 2.30**
Insulin (pmol/l)	104.64 ± 20.93	517.96 ± 156.96^*^

Data are presented as mean ± SEM and are representative of five or six mice/group (control [WT] three male and two female mice; *db*/*db* three male and three female mice)

Body weight, glucose levels and insulin levels were significantly increased in *db*/*db* mice when compared with control mice at 14 weeks of age

^**^p<0.001 vs control; ^*^p=0.033 vs control

### Actimetry and new object discrimination task

Episodic memory for ‘what,’ ‘where’ and ‘when’ paradigms was analysed, following procedures shown in ESM Methods. ‘What’ was the difference in time exploring familiar and recent objects, ‘where’ was the difference in time exploring displaced and non-displaced objects and ‘when’ was the difference between time exploring familiar non-displaced and recent non-displaced objects. All measurements were performed in triplicate and data were expressed as percentage of control values. Discrimination index was calculated as the percentage of time exploring recent objects/total exploration time.

### Morris water maze

Spatial learning and memory abilities were analysed in the Morris water maze (MWM) (see ESM Methods). Time required to locate the platform in the acquisition phase, number of entrances in quadrant 2 and swimming velocity during the retention phase were analysed using Smart software version 3.0.04 (Panlab S.L.U., Barcelona, Spain).

### Tissue dissociation

For scRNA-seq, one male homozygous (*db/db*) mouse and one male heterozygous (*db/+)* mouse were killed at 16 weeks of age. Whole cortex was dissected and dissociated using the papain dissociation system (Worthington, Lakewood, NJ, USA). Cortex samples were digested in papain (20 U/ml) for 45 min at 37°C. After treating with ovomucoid, cells were re-suspended in PBS with 0.04% wt/vol. BSA. Cell counts and viability was checked using Trypan Blue (Sigma-Aldrich, Missouri, USA).

### scRNA-seq library preparation, sequencing and associated analysis

scRNA-seq libraries were generated using the 10x Genomics Chromium Single Cell 3′ Reagent Kit v3 and sequenced on Illumina NovaSeq6000 S2 200 flow cell at the Genomics Core Technology Unit (GCTU), Queen’s University Belfast. Analysis was completed as discussed in ESM Methods using Seurat R package v.4.2.0 (https://satijalab.org/seurat/; developed by P. Hoffman, S. Lab and collaborators, New York City, NY, USA).

### Identification of cell types, marker genes and differential expression analysis

Following procedures detailed in ESM Methods, cortical cell types were identified by expression of established markers (Table 2). Differential gene expression, single-cell regulatory network, weighted gene correlation network (WGCN) [17] and pseudotime trajectory [18] analyses were performed.

**Table 2:** Marker genes used for identification of cell types

Cell type	Marker gene	Gene name
Mature neurons	*Syt1*	Synaptotagmin 1
Immature neurons	*Sox11*	SRY-box transcription factor 11
Inhibitory neurons	*Gad1*, *Gad2*	Glutamate decarboxylase 1, 2
Astrocytes	*Gja1*	Gap junction protein α1
Oligodendrocytes	*Cldn11*	Claudin 11
Oligodendrocyte precursor cells	*Pdgfrb*	Platelet-derived growth factor β
Microglia	*Tmem119*	Transmembrane protein 119
Endothelial	*Cldn5*	Claudin 5
Pericytes	*Kcnj8*	Potassium inwardly rectifying channel subfamily J member 8
Vascular smooth muscle cells	*Slc6a13*	Solute carrier family 6 member 13
Choroid plexus cells	*Ttr*	Transthyretin

### Tissue preparation and staining

Immediately after mice were killed via overdose of pentobarbital, *db/db* and control mouse brains were harvested and weighed. Left hemispheres were fixed in 4% wt/vol. paraformaldehyde for 1 week and 30 µm coronal brain sections were obtained in a Microm HM450 microtome (ThermoFisher, Spain). Sections were stored in 50% wt/vol. glycerol at −20°C until used. For staining procedures, consecutive sections were selected at 1.5, 0.5, −0.5, −1.5, −2.5 and −3.5 mm from Bregma. Staining was carried out for neuronal nuclear protein (NeuN) [19], BrdU and doublecortin (DCX) [20] as previously described.

### Cresyl violet staining

Sections were processed as described [21] (see ESM Methods). Images were acquired using an optical Olympus Bx60 microscope with an attached Olympus DP71 camera (Olympus, Tokyo, Japan) and mmi CellTools software version 4.3 (Molecular Machines & Industries, Eching, Germany). Hemisection size and cortical thickness were measured using Adobe Photoshop Elements 2.0 version 6.2 (Adobe, USA) and Image J version 1.49 (National Institutes of Health, MD, USA) software.

### Iba1 and GFAP staining

Sections were pre-treated with Tween-20 (BP337; Fisher Scientific, UK) (0.05%) (wt/vol.) in citrate buffer (10 mmol/l) for 2 h at 65°C and blocked in 3% (wt/vol.) BSA (A7906; Sigma-Aldrich, Madrid, Spain) and 0.5% (wt/vol.) Triton X100 (BP151; Fisher Scientific) for 1 h. Sections were incubated with anti-Iba1 (ionised calcium binding adaptor molecule 1) (ESM Table 1) and anti-GFAP (glial fibrillary acidic protein) (ESM Table 1) antibodies overnight at 4°C in 3% (wt/vol.) BSA and 0.5% (wt/vol.) Triton X100 followed by incubation with secondary antibodies (diluted in PBS) (ESM Table 1) for 1 h at room temperature.

### Vascular staining

Antigen retrieval was performed using citrate buffer at 95°C for 30 min in a water bath. Slides were permeabilised in 2% (wt/vol.) Triton-X for 30 min. Slides were blocked in 5% donkey serum in 0.2% (wt/vol.) Triton-X for 1 h at room temperature. Primary antibodies (ESM Table 1) were added and slides were incubated overnight at 4°C in 0.5% donkey serum and 0.2% Triton-X followed by incubation with secondary antibodies (ESM Table 1) for 2 h at room temperature, in PBS.

### Image analysis

Images were taken via Olympus DP71 camera or Leica SP8 confocal. Counting of cell populations was performed by selecting 15–20 regions of interest (ROIs) (7960.215 µm^2^/ROI) from each cortical section (333–417 ROIs/group) and counting using Image J Software version 1.49 (National Institutes of Health, MD, USA).

### Metabolic assessment of cortical brain punches

The method published by Qi et al (2021) [22] was adapted in order to assess glycolysis in acute coronal slices from the cortex of five male *db/db* and five male *db/+* mice, as described in ESM Methods. The ‘glycolysis stress test’ protocol from Agilent (USA) was optimised for use with cortex slices (ESM Table 2). Protocol length and injection timings were as follows: baseline, 4 cycles; glucose injection, 5 cycles; oligomycin A injection, 5 cycles; and 2-deoxy-d-glucose (2-DG), 10 cycles. Each cycle consisted of 3 min mix, 1 min wait, 2 min measure. Analysis of basal glycolysis, glycolytic capacity and glycolytic reserve was performed in Wave Software version 2.6.3.5 (Agilent).

### Human tissue

For IHC staining, post-mortem brain samples of the frontal cortex of eight individuals were provided by the Netherlands Brain Bank, Amsterdam. The brain samples were from donors with or without diabetes mellitus. Classification of the donors was based on clinical diagnosis and confirmed by autopsy. The staining procedures were performed as described in ESM Methods. Antibody information is included in ESM Table 3.

For western blotting, post-mortem brain samples were obtained as frozen pieces of cortical tissue from the neurological tissue biobank of Hospital Clínic – IDIBAPS in Barcelona, Spain. Control donors did not have signs of neurological alterations. Samples from individuals with type 2 diabetes clinically diagnosed with dementia were excluded from the study. The most relevant associated comorbidities are presented in ESM Table 4. Proteins were extracted and western blots were run as described in ESM Methods. Antibody information is included in ESM Table 3.

### Analysis and statistics

Randomisation and blinding at the experimental phase was not possible due to the obese nature of *db/db* mice. Quantification and analysis of mouse data was done by two independent, blinded researchers. Student’s *t* test for independent samples, or Mann–Whitney test was used to compare control and *db*/*db* mouse groups. Two-way ANOVA (group × session) was used to analyse the acquisition phase in the MWM. IBM SPSS Statistics software version 29.0.0.0 (IBM Corporation, NY, USA) was used for all statistical analysis and results are expressed as mean ± SEM.

### Study approval

The Animal Care and Use Committee of the University of Cadiz, in accordance with the Guidelines for Care and Use of experimental animals (European Commission Directive 2010/63 /UE and Spanish Royal Decree 53/2013), approved all experimental procedures. The Animal Welfare Ethical Review Body at Queen’s University Belfast approved this study, and all procedures were conducted under the regulation of the UK Home Office Animals Scientific Research Act 1986. The collection of the human brain material for immunostaining was done in accordance with Dutch laws and the rules outlined by the local ethics committee. For western blotting, samples were obtained by the biobank after corresponding ethical approvals and following the regulations of local ethics committees in Universitat de Barcelona, and in accordance with the Helsinki Declaration and the Convention of the Council of Europe on Human Rights and Biomedicine.

## Results

### ***db*/*db* mice exhibit altered memory function and reduced cortical thickness**

**Fig. 1:**
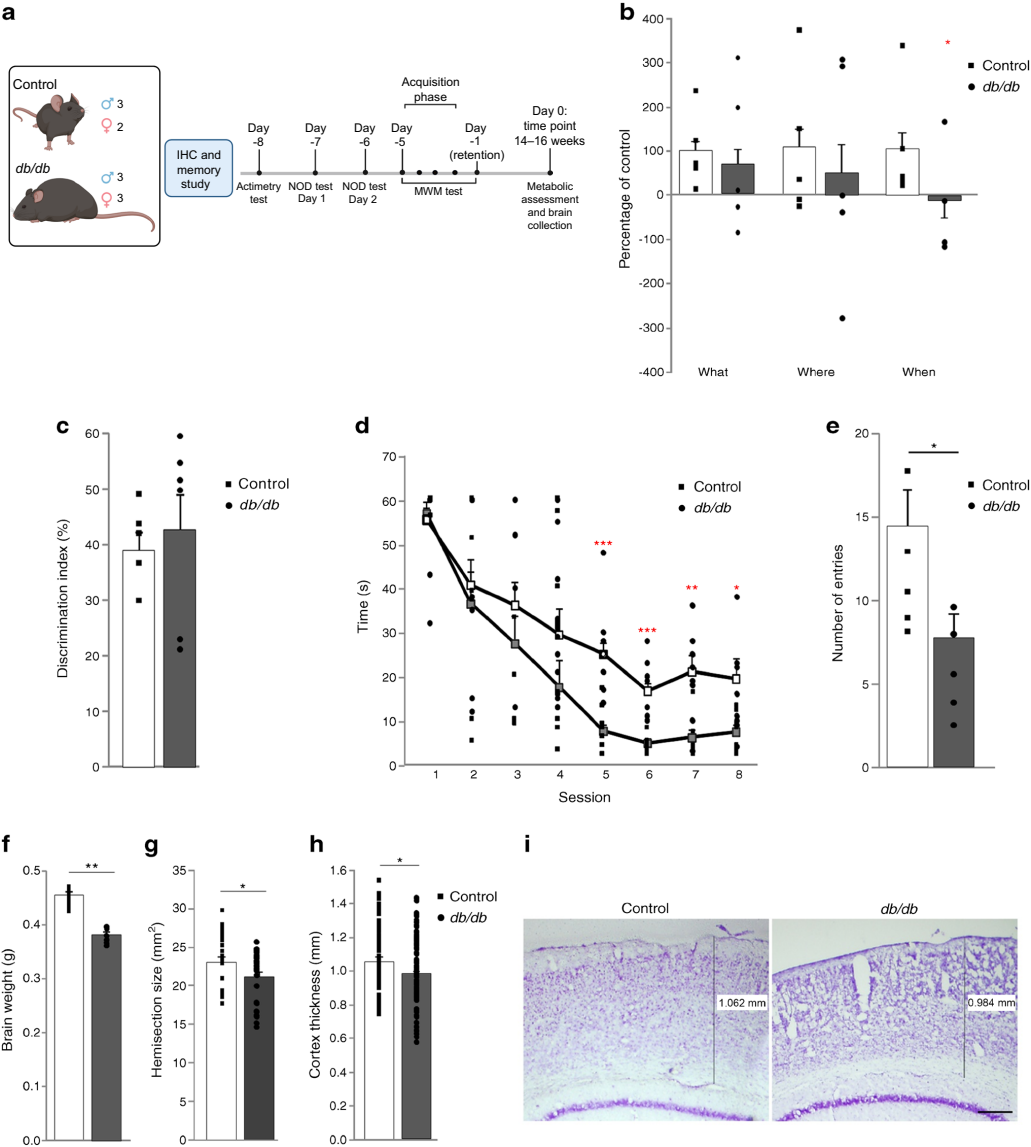
Cognitive impairment and brain atrophy in *db/db* mice. (**a**) Schematic detailing study design and time points. (**b**) The new object discrimination test showed that episodic memory was not affected in *db/db* mice when ‘what’ (*p*=0.441) or ‘where’ (*p*=0.484) paradigms were analysed. However, the ‘when’ paradigm was significantly impaired in *db*/*db* mice (*p=0.016). Data are representative of five mice. (**c**) Discrimination index was not affected in *db*/*db* mice (*p*=0.530). Data are representative of five or six mice. (**d**) Learning impairment was observed in *db*/*db* mice in the acquisition phase of the MWM. Times required to locate the platform were significantly longer in *db*/*db* mice than control mice in sessions 5–8 (session 1 *p*=0.729, session 2 *p*=0.637, session 3 *p*=0.294, session 4 *p*=0.168, session 5 ***p<0.001 vs control, session 6 ***p<0.001 vs control, session 7 **p=0.003 vs control, session 8 *p=0.025 vs control). Data are representative of five or six mice. White squares, mean value for *db/db* mice; grey squares, mean value for control mice. (**e**) The number of entries into quadrant 2 (where the platform was located along the acquisition phase) was significantly lower in *db*/*db* mice (*p<0.05). (**f**) Brain weight was reduced in *db*/*db* mice when compared with control mice (**p<0.001 vs control). (**g**) Hemisection size was significantly reduced in *db*/*db* mice (*p=0.039 vs control). (**h**) Cortical thickness was significantly reduced in *db*/*db* mice (*p=0.013 vs control). (**i**) Representative images showing reduced cortical thickness in *db*/*db* mice. Scale bar, 250 µm. Schematic in (**a**) was created with BioRender.com

The *db/db* mouse displays key characteristics of type 2 diabetes including obesity, hyperglycaemia and hyperinsulinaemia (Table 1) [21, 23]. A schematic of the study is shown in Fig. 1a.

Episodic memory was compromised in *db/db* mice (Fig. 1b). While no differences were detected for ‘what’ or ‘where’ paradigms, a significant impairment was observed for ‘when’ paradigm. A similar discrimination index was observed when comparing the groups of mice (Fig. 1c). Assessment of spatial memory using the MWM revealed no significant group × session effect (*F*_(1,7)_=1.009, *p*=0.427), although individual session assessment revealed that *db/db* mice had great difficulty in learning the location of the hidden platform. This was reflected from session 5 onwards (Fig. 1d). The number of entries into the quadrant where the platform used to be located was significantly lower in *db/db* mice (Fig. 1e). Swimming speed was reduced in *db/db* mice (ESM Table 5) and while this may impact the results of the MWM, we have also shown that walking speed (and therefore motor activity) is not compromised in these mice. At the time of death, brain weight was significantly lower in *db/db* mice (Fig. 1f) [24]. Cresyl violet staining revealed that hemisection sizes were also significantly smaller in *db/db* mice (Fig. 1g). *db/db* mice presented significantly thinner cortices when compared with control mice (Fig. 1h,i).

### Single-cell transcriptome of ***db/db*** mouse cortex reveals massive changes in identity and composition of various key cell types

**Fig. 2:**
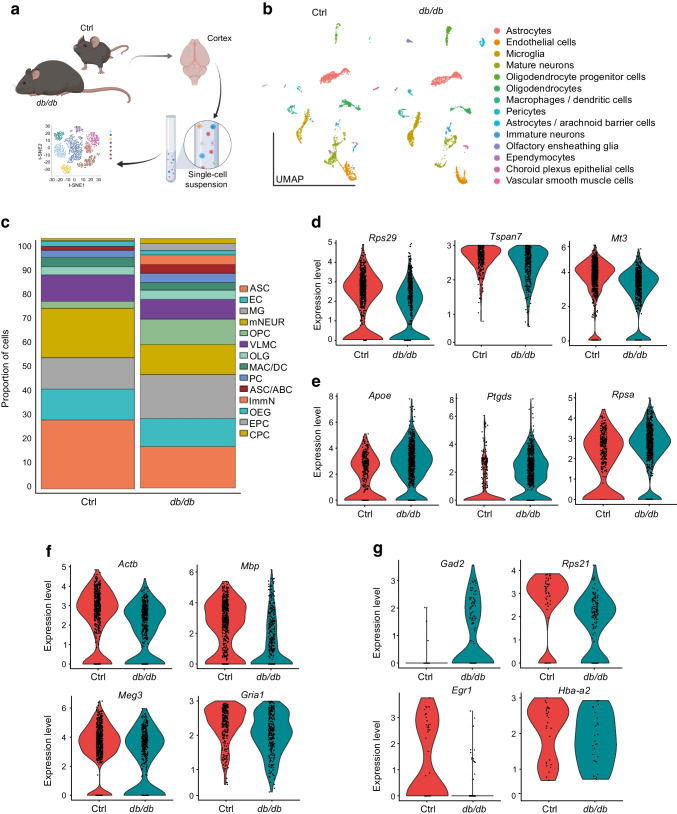
Single-cell sequencing of diabetic mouse brain reveals changes in glial and neuronal populations. (**a**) Schematic of scRNA-seq data derivation. *db*/*db* and *db*/*+* (control) mouse cortex was dissociated in papain when mice were 16 weeks old. The resulting single-cell solution was sequenced via 10× genomics and analysis was performed in R. (**b**) Clustering of the cortex showed similar cell populations in diabetic and control mouse brain. (**c**) Proportion of each cell type in *db*/*db* and *db*/*+* mouse brain cortex, indicating the difference in astrocytes, microglia and neuronal populations. (**d**–**g**) Significant differential expression of genes (all *p*<0.001) in astrocytes (**d**), microglia (**e**), mature neurons (**f**) and immature neurons (**g**). Expression levels for individual genes are presented as the normalised counts. Schematic in (**a**) was created with BioRender.com. ABC, arachnoid barrier cells; ASC, astrocytes; CPC, choroid plexus cells; Ctrl, control; DC, dendritic cells; EC, endothelial cells; EPC, ependymocytes; ImmN, immature neurons; MAC, macrophages; MG, microglia; mNEUR, mature neurons; OEG, olfactory ensheathing glia; OLG, oligodendrocytes; OPC, oligodendrocyte progenitor cells; PC, pericytes; VLMC, vascular leptomeningeal cells

Single-cell transcriptome analysis of cortex from *db/db* mice revealed significant changes in neuronal and glial populations (Fig. 2a,b). There was a marked increase in the microglia population of the *db/db* mouse cerebral cortex accompanied by a loss of astrocytes and mature neurons (Fig. 2c). An increased fraction of immature neurons was observed, alongside an increase in oligodendrocyte progenitors and concomitant decrease in mature oligodendrocytes (Fig. 2c). Pseudotime trajectory on glia populations (astrocytes and oligodendrocytes together) (ESM Fig. 1) showed cellular heterogeneity and an altered lineage trajectory in *db*/*db* vs control mice. These results suggest altered neuronal development, glial development and maturation trajectory in the *db/db* mouse cortex.

### Molecular analysis of cortical cell populations reveals critical changes in diabetic mouse brains

**Fig. 3:**
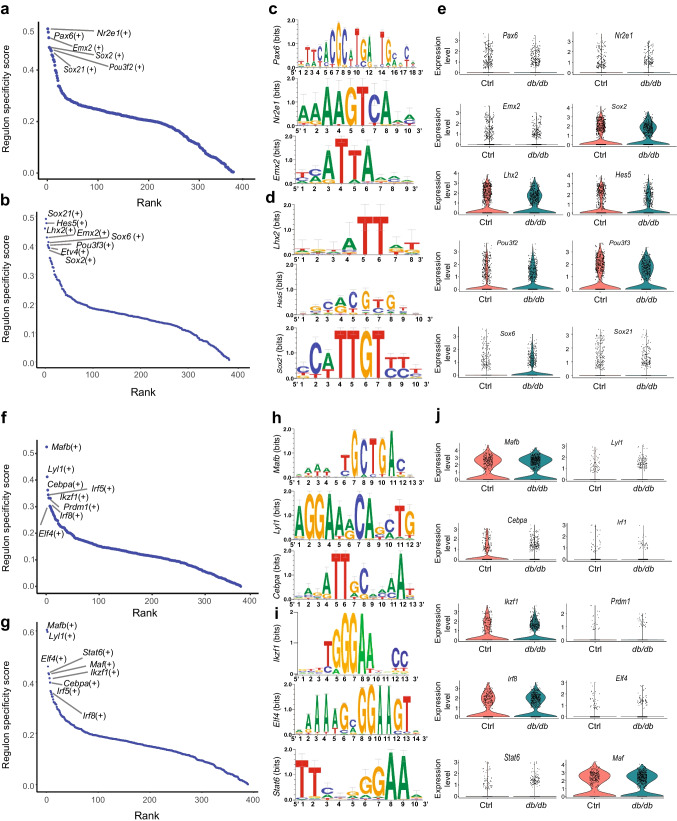
Regulon analysis. (**a**–**d**) Top regulons (**a**, **b**) and top unique motifs (**c**,** d**) in *db*/+ (**a**, **c**) and *db*/*db* (**b**, **d**) mouse astrocytes. (**e**) Differential expression patterns of top regulon genes in *db*/+ and *db*/*db* mouse astrocyte cell populations. (**f**–**j**) Top regulons (**f**, **g**) and unique motifs (**h**, **i**) in *db*/+ (**f**, **h**) and *db*/*db* (**g**, **i**) mouse microglia. (**j**) Differential expression patterns of top regulon genes in *db*/+ and *db*/*db* mouse microglia cell populations. Expression levels for individual genes are presented as the normalised counts. Neuronal populations are shown in ESM Fig. 2

Focused molecular analysis on neuronal and glial specific transcripts (Fig. 2d–g) demonstrated that genes encoding ribosomal protein S21 and S29 (*Rps21* and *Rps29*, respectively) were downregulated in immature neurons as well as astrocytes, with upregulation of ribosomal protein SA (encoded by *Rpsa*) in microglia. Transcripts linked to inflammation and microglial activation were altered in *db/db* mouse microglia: expression of genes encoding apolipoprotein E (*Apoe*) [25] and prostaglandin D2 synthase (*Ptgds*) [26] was upregulated. Furthermore, downregulated genes in mature neurons were major mediators and regulators of synaptic neuronal functions such as the genes encoding glutamate ionotropic receptor AMPA type subunit 1 (*Gria1*) [27] and myelin basic protein (*Mbp*) [28]. Mature neurons showed downregulation of genes encoding maternally expressed gene 3 (*Meg3*) and β-actin (*Actb*). Astrocytes displayed downregulation of actin polymerisation regulating genes tetraspanin 7 (*Tspan7*) [29] and metallothionein III (*Mt3*) [30]. Immature neurons showed downregulation of the gene encoding early growth response-1 (*Egr1*), an important transcription factor in memory formation [31]. This occurred alongside upregulation of *HbA-a2* (encoding a globin family protein) and *Gad2*, which are known to be linked to schizophrenia and bipolar disorder [32]. Analysis by the algorithm pySCENIC identified enhanced expression of the transcription factor genes (*Hes5*, *Lhx2*, *Sox21*, *Sox2*) and neurogenesis regulatory factors in *db/db* mouse astrocytes (Fig. 3a–e), suggesting a potentially ambiguous lineage trajectory between *db/db* and control mice. Top microglial regulons were slightly discrete between control and *db/db* mice (Fig. 3f–j). The mature neuronal population (*db/db*) showed top regulons that are known transcription factors (*Elk1* and *Egr4*) regulating early immediate genes (ESM Fig. 2a–e), while immature neuronal cells (*db/db*) had increased expression of marker genes for inhibitory neuronal characteristics (*Sp9* and *Meis1*) (ESM Fig. 2f–j). These outcomes suggest a shift in the gene regulatory networks and driver transcription factors underlying distinct cell fates in *db/db* mouse cortex. The corresponding scale independence and mean connectivity are shown in ESM Fig. 3.

### Ex vivo investigation validates neuronal and glial population alterations in ***db/db*** mice

**Fig. 4:**
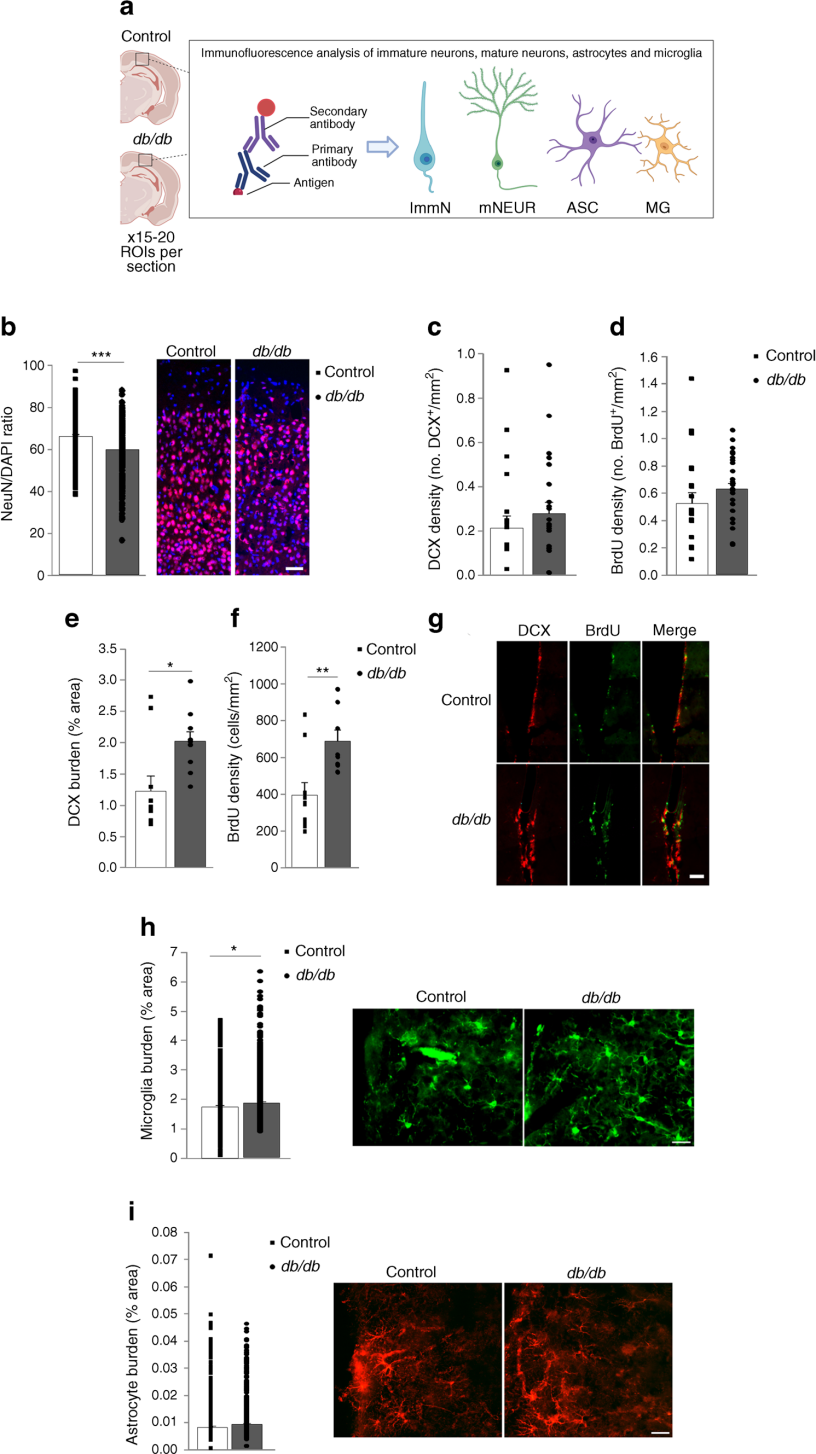
Neuron compromise and microglia burden are increased in *db*/*db* mice. (**a**) Schematic describing the staining process. Indirect staining with primary antibody and secondary fluorescently conjugated antibody was used to identify cell subtypes (immature neurons, mature neurons, astrocytes and microglia). (**b**) NeuN/DAPI ratio was significantly lower in the cortex from *db*/*db* mice, when compared with control mice (***p<0.001 vs control). Representative images of cortical sections from control and *db*/*db* mice, with NeuN (red) and DAPI (blue) staining, are shown. Scale bar, 50 µm. (**c**, **d**) No differences were observed when DCX (*p*=0.407) (**c**) or BrdU (*p*=0.267) (**d**) densities were compared in the cortex from *db*/*db* and control mice. (**e**, **f**) DCX burden (**e**) was significantly increased in the SVZ from *db*/*db* when compared with control mice (*p=0.012 vs control). Similarly, BrdU density (**f**) was significantly higher in the SVZ from *db*/*db* mice (**p=0.004 vs control). (**g**) Representative images of the SVZ from control and *db*/*db* mice (green, BrdU^+^; red, DCX^+^). Scale bar, 50 μm. (**h**) Microglia burden was increased in *db*/*db* mice when compared with control mice (*p=0.046). Representative images of microglia staining in the cortex from control and *db*/*db* mice (green, Iba-1) are shown. Scale bar, 25 μm. (**i**) Astrocyte burden was not affected in *db*/*db* mice when compared with control mice (*p*=0.142). Representative images of astrocyte staining in the cortex from control and *db*/*db* mice (red, GFAP) are shown. Scale bar, 25 μm. Schematic in (**a**) was created with BioRender.com. ASC, astrocytes; ImmN, immature neurons; MG, microglia; mNEUR, mature neurons

Immunohistochemistry (Fig. 4a) was used to quantify the numbers of immature and mature neurons (Fig. 4b–g). NeuN^+^ cell/DAPI ratio was significantly reduced in *db/db* mice (Fig. 4b). A slight increase in the density of DCX^+^ and proliferating cells was observed in *db/db* mice (Fig. 4c,d). Since the cortex is not a classical neurogenic niche, proliferation and neurogenesis were also assessed in the subventricular zone (SVZ). Both DCX burden and BrdU^+^ cell density in the SVZ were significantly increased in *db/db* mice when compared with control mice (Fig. 4e–g).

Microglia burden was increased in the cortex from *db/db* mice when compared with control mice (Fig. 4h), while no differences in astrocyte burden were observed (Fig. 4i).

### Correlated gene modules reveal the altered cellular behaviour of metabolism, synapse and inflammatory pathways in ***db/db*** mouse cortex

**Fig. 5:**
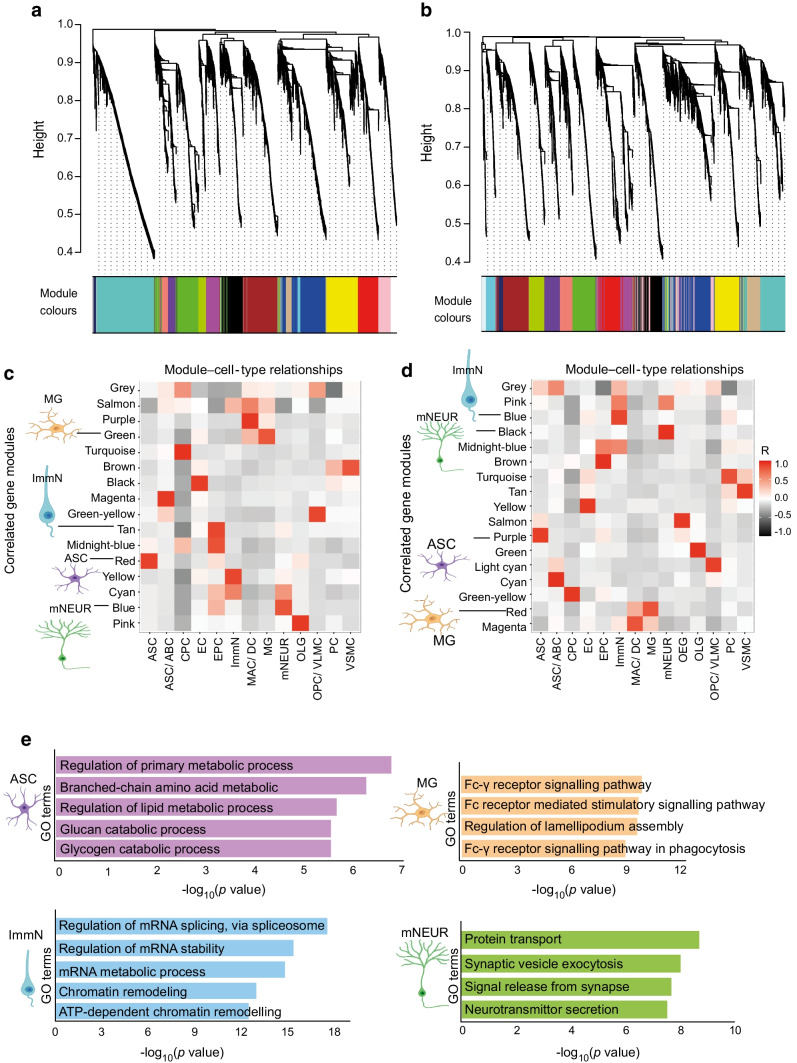
WGCN analysis and GO terms for correlated modules. (**a**, **b**) Cluster dendrogram for gene modules showing modules of high interconnection in control (**a**) and *db/db* (**b**) mice. Height in cluster dendrograms indicates the inter-cluster correlation distance and modules are marked by different colours in the horizontal bar (grey represents unassigned genes). (**c**, **d**) The relationship between gene sets and cell types is shown for control (**c**) and *db/db* (**d**). Each column is a cell type and each row represents module eigengenes. Each unit is coloured in the correlation coefficient (*R*) on a scale of −1 to +1 (black, negative; red, positive; white, null). (**e**) Unique GO terms for correlated gene module in *db*/*db* mouse cell types. Cell images were created with BioRender.com. ABC, arachnoid barrier cells; ASC, astrocytes; CPC, choroid plexus cells; DC, dendritic cells; EC, endothelial cells; EPC, ependymocytes; ImmN, immature neurons; MAC, macrophages; MG, microglia; mNeur, mature neurons; OLG, oligodendrocytes; OPC, oligodendrocyte progenitor cells; PC, pericytes; VLMC, vascular leptomeningeal cells; VSMC, vascular smooth muscle cells

WGCN analysis was performed (Fig. 5a–d). Gene ontology (GO) analysis of correlated unique gene modules in cells revealed altered mRNA metabolism in immature neurons and changes in glycogen and lipid metabolism in astrocytes of diabetic mouse brains (Fig. 5e). Furthermore, pathways implicated in inflammation and phagocytosis in microglia were uniquely altered in diabetic cortex, whereas mature neurons had altered pathways relating to synaptic processes (Fig. 5e).

### Vascular abnormalities observed in ***db/db*** mouse cortex

**Fig. 6:**
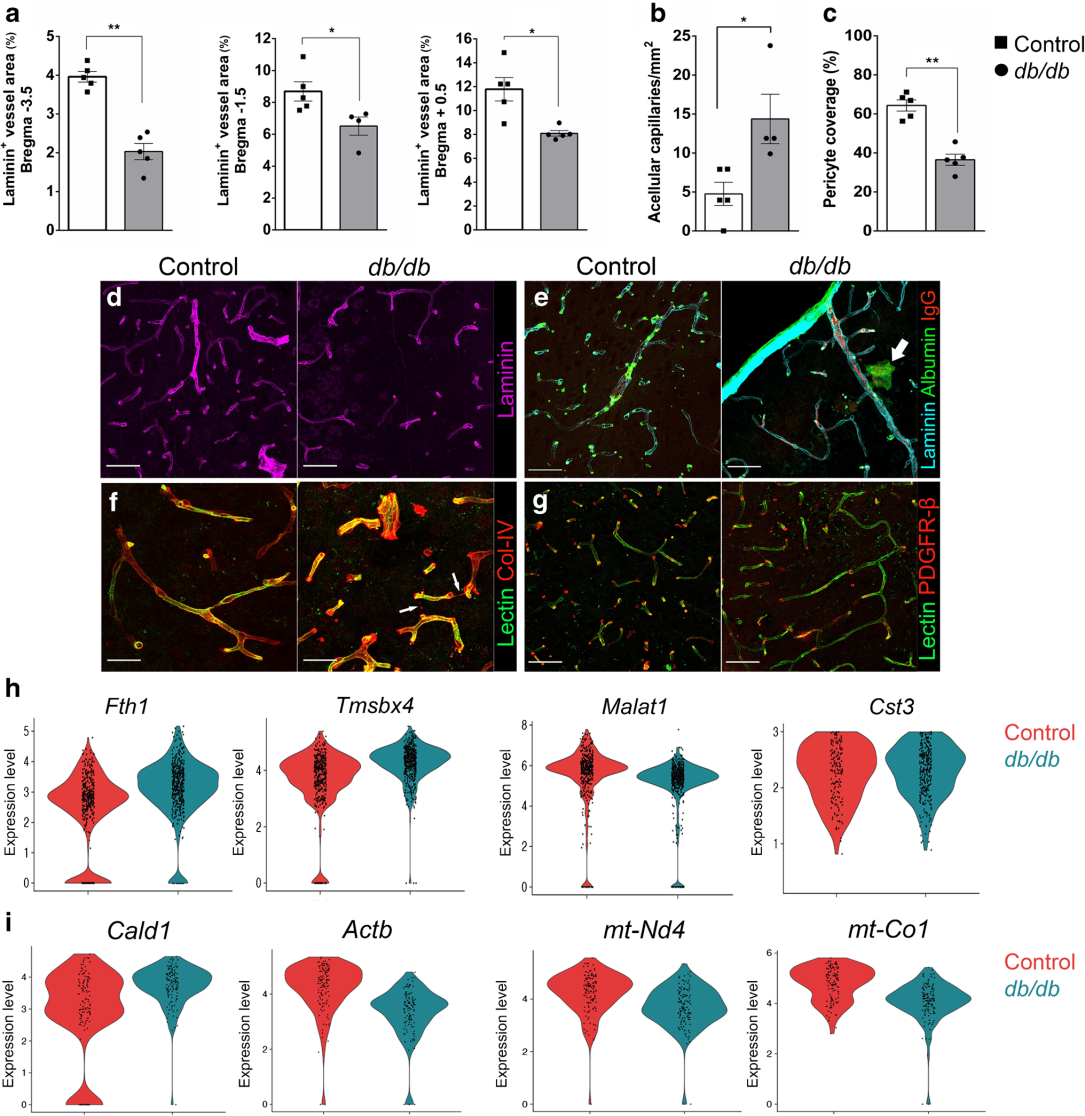
Vascular alterations in *db*/*db* mouse cortex. (**a**) Laminin^+^ vessel area was significantly reduced at various levels of Bregma in the cortex of *db*/*db* mice (*p<0.05, **p<0.01). (**b**) A significant increase in acellular capillaries was observed in *db*/*db* mouse cortex (*p<0.05) at Bregma level +0.5 mm. (**c**) A significant loss of pericyte coverage (PDGFR-β) was observed in *db*/*db* mouse cortex at Bregma +0.5 mm (**p<0.01). (**d**–**g**) Representative images: laminin staining (purple) (**d**); ‘hot-spot’ (arrow) area of vessel leakage in *db*/*db* mouse cortex (blue, laminin; red, IgG; green, albumin) (**e**); lectin (green) and Col-IV (red) in control and *db*/*db* mouse cortex for assessment of acellular capillaries (arrows) (**f**); and lectin (green) and PDGFR-β (red) in the control and *db*/*db* mouse cortex for measurement of pericyte coverage (**g**). Scale bar, 50 µm. (**h**) DEGs in endothelial cells. (**i**) DEGs in pericytes. Expression levels for individual genes (all *p*<0.001) are presented as the normalised counts

Previous reports have suggested that brain vasculature is affected in type 2 diabetes models [24]. We observed reduced laminin^+^ vessels in *db/db* mice (Fig. 6a, d). ‘Hotspots’ of leakage were observed in the *db/db* mouse cortex but not in the control mice cortex (Fig. 6e). Expression of endothelial marker genes including *Lgals1* (encoding galectin 1) and *Lamc3* (encoding laminin γ subunit 3) was reduced in *db/db* mice (ESM Fig. 4). The top differentially expressed genes (DEGs) in *db/db* mouse endothelial cells included *Malat* (encoding metastasis-associated lung adenocarcinoma transcript 1), which may be involved in angiogenesis [33], and *Cts3*, which is dysregulated in cerebral amyloid angiopathy (Fig. 6h) [34]. There was an increase in acellular capillaries in the *db/db* mouse cortex (Fig. 6b,f) alongside a loss of pericyte coverage (Fig. 6c,g). The top DEGs in pericytes included *Cald1* (encoding downregulation of caldesmon 1), which is involved in smooth muscle contraction [35]. Dysregulation of metabolic genes was observed in *db/db* mouse pericytes, with a loss of mitochondrial complex-related genes such as *mt-Co1* and *mt-Nd4* (encoding mitochondrial NADH dehydrogenase 4) (Fig. 6i).

### Cortical metabolism is significantly altered in ***db/db*** mice, with dysregulated metabolic pathway genes observed in specific cortical populations

**Fig. 7:**
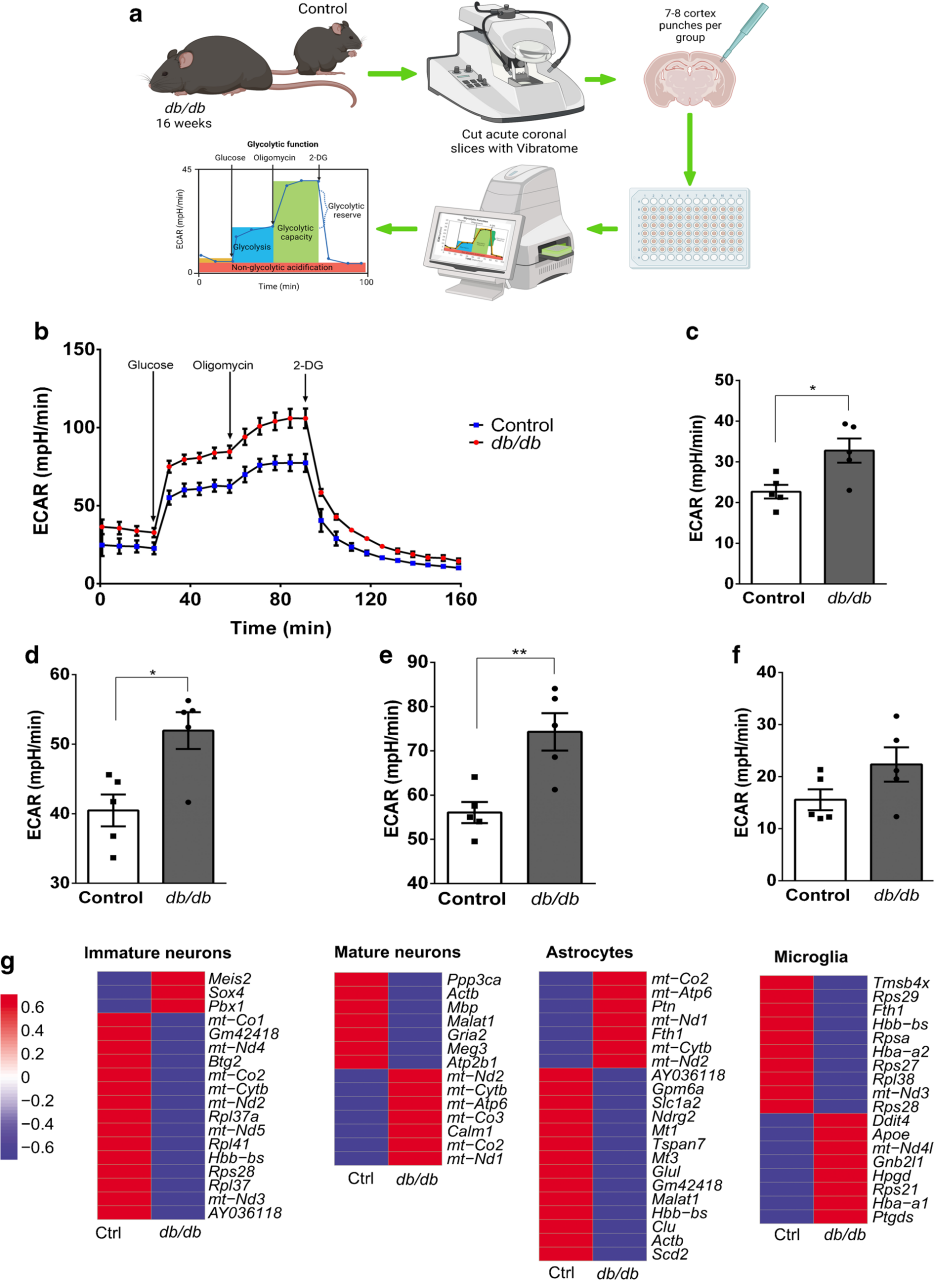
Metabolic profiling of *db*/*db* mouse cortex. (**a**) Schematic describing the process of obtaining acute coronal sections for metabolic profiling by Seahorse X FE 96. Male *db*/*db* or control mice aged 16 weeks (*n*=5 mice per group) were used to generate acute coronal slices (250 µm) using the Leica VT100s Vibratome. The Seahorse analyser was used to assess glycolytic function, via an adapted glycolytic stress test protocol. (**b**) Traces from *n*=5 *db/db* and *n*=5 control mice (representative of seven or eight punches per mouse) following the adapted glycolysis stress test protocol. (**c**) Non-glycolytic acidification was significantly increased in *db*/*db* mice. (**d**) Basal glycolysis was significantly increased in *db*/*db* mice. (**e**) Glycolytic capacity was significantly increased in *db*/*db* mice. (**f**) No significant changes were observed between *db*/*db* and control mice when comparing glycolytic reserve. *n*=5 mice per group; seven or eight cortical punches per mouse. *p<0.05, **p<0.01. Unpaired Student’s *t* test. Data are presented as mean values ± SEM. (**g**) Metabolism-related DEGs (all *p*<0.001) in *db*/+ (control) and *db*/*db* mouse cortex. Heatmap depicts normalised expression counts scaled between +1 and −1. Schematic in (**a**) was created with BioRender.com. ECAR, extracellular acidification rate

Our results indicated that metabolic dysfunction in the type 2 diabetes brain may be a consequence of NVU impairment. To validate these findings in vivo, metabolic profiling of the cortex was performed using acute brain slices (Fig. 7a). Glycolytic function was increased in *db/db* mouse cortex compared with controls (Fig. 7b). Non-glycolytic acidification (Fig. 7c), basal glycolysis (Fig. 7d) and glycolytic capacity (Fig. 7e) were all significantly increased in the *db/db* mouse cortex; however, no significant difference was observed in glycolytic reserve (Fig. 7f).

A number of metabolism-related genes were differentially expressed in the *db/db* mouse cortex (Fig. 7g). For example, astrocytes and mature neurons showed increased expression of electron transport chain subunit genes and NADH dehydrogenase subunits. Together, these data indicate that the metabolic processes of cortical NVU cell populations are altered in *db/db* mice.

### Cortical cell populations are also changed in humans with type 2 diabetes

**Fig. 8:**
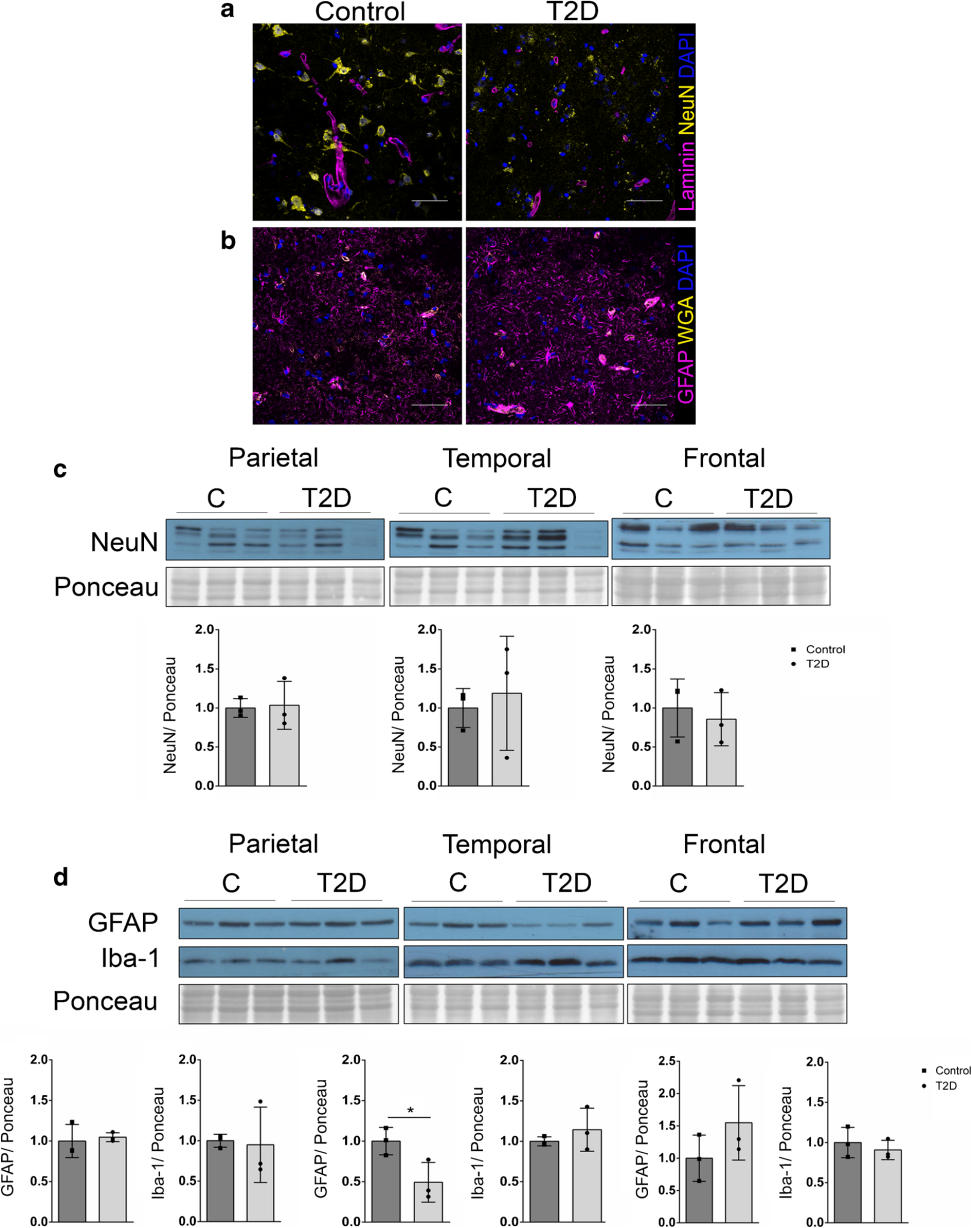
Cortex samples from individuals with type 2 diabetes reveal alterations in neuronal and glial cell populations. (**a**) Representative images show reduced NeuN staining (yellow) in frontal cortex from a donor with type 2 diabetes compared with cortex from a control donor. Laminin staining is shown in purple, alongside DAPI in blue, *n*=4 or 5 individuals per group. (**b**) Representative images showing GFAP staining of the frontal cortex (purple) alongside wheat germ agglutinin (yellow), *n*=4 or 5 patients per group. (**c**) Western blotting of human cortex samples (parietal, temporal and frontal) shows the expression of NeuN in each location, *n*=3 individuals per group. (**d**) Western blotting of human cortex samples (parietal, temporal and frontal) shows the expression of GFAP and Iba-1 in each location. GFAP expression was reduced in the temporal cortex (*p<0.05 vs control samples), *n*=3 individuals per group. Western blot quantification was analysed by normalising the protein levels to the Ponceau stain. C, control; T2D, type 2 diabetes; WGA, wheat germ agglutinin

To validate our *db/db* mouse model findings, we studied samples from people with type 2 diabetes. Neuronal and glial cell populations were assessed in a small population of cortex samples from non-diabetic individuals and from individuals with type 2 diabetes. Staining of NeuN in frontal cortex sections indicated a qualitative reduction in neuronal cells in the frontal cortex of individuals with type 2 diabetes (Fig. 8a), although semi-quantitative analysis of NeuN western blots from parietal, temporal and frontal cortex samples showed no significant difference (Fig. 8c). A similar observation was seen after analysis of postsynaptic density protein 95 (PSD95) (ESM Fig. 5a). Noticeably, a reduction in GFAP protein was observed in samples from type 2 diabetes temporal cortex only (Fig. 8b,d). GFAP staining of human cortex showed an increased localisation with blood vessels (stained with wheat germ agglutinin) (Fig. 8b). Iba-1 was not significantly changed in any cortical region in individuals with type 2 diabetes when compared with healthy control individuals. A reduction in zonula occludens-1 (ZO-1) protein levels was observed in individuals with type 2 diabetes in the parietal and temporal cortex (ESM Fig. 5b). Occludin was unexpectedly increased in the parietal cortex of individuals with type 2 diabetes (ESM Fig. 5b). A statistically significant loss of platelet-derived growth factor receptor (PDGFR) α/β was observed in the parietal cortex of individuals with type 2 diabetes (ESM Fig. 5c).

## Discussion

Recent evidence has shown a relationship between type 2 diabetes and cognitive decline, dementia and Alzheimer’s disease [2-4]. Hyperglycaemia, insulin resistance, hypertension and dyslipidaemia in type 2 diabetes have a profound effect on the vasculature, leading to complications such as stroke, cardiovascular disease and diabetic retinopathy. Type 2 diabetes also has a damaging effect on neurons and glia, often relating to cell–cell communication defects across the retinal NVU [12]. The current study has detailed how *db/db* mice manifest cognitive deficiencies in unison with reduced brain weight and cortex size. Here, for the first time, we have catalogued a map of transcriptional profiles of the cerebral cortex from *db/db* mice, with a focus on the cell populations of the NVU.

Our scRNA-seq analysis identified a decrease in mature neurons, alongside an increase in microglia and immature neurons in the *db/db* mouse cortex. These novel findings, including disruption of several key neuronal regulatory genes, were complemented by the observed brain atrophy and cognitive decline in the *db/db* mouse. *Meg3* and *Mbp* both showed reduced expression in *db/db* mice, indicating functional disruption. Downregulation of *Mbp* in mature neurons may indicate that golli-Mbp [28] (a known protein found outside the myelin sheath), which is involved in neuronal development [36] and Ca^2+^ homeostasis, may be affected in type 2 diabetes. We also observed some evidence of NeuN reduction in individuals with type 2 diabetes. Together, these data suggest that type 2 diabetes may affect neuronal maturation, thereby having a profound impact on cognition. This finding is reinforced by similar findings in Alzheimer’s disease [37] and other neurodegenerative diseases associated with progressive cognitive decline [38].

Previous reports have suggested that glial GFAP^+^ area is increased in the *db/db* mouse cortex [39]. Our findings in *db/db* mice and humans with type 2 diabetes remain ambiguous. Nevertheless, we observed a distinct transcriptional profile of cortical astrocytes in *db/db* mice. A notable example is *Mt3* downregulation in *db/db* mouse astrocytes. This change in expression has been related to reduced uptake of Aβ leading to extracellular accumulation in Alzheimer’s disease [40], highlighting a link between Alzheimer’s disease pathology and diabetes-induced cognitive decline. *Mt1* and *Mt3* are also involved in protection against oxidative stress [30] and their downregulation in *db/db* mouse astrocytes may contribute to oxidative damage and enhanced inflammation in the type 2 diabetes cortex.

Cortical astrocyte metabolism was affected in *db/db* mice, including upregulation of genes involved in the formation of mitochondrial complexes (e.g. *mt-Co2*, *mt-Nd1*, *mt-Nd2*) and dysregulated lipid metabolism. A previous study reported that alterations in astrocyte lipid metabolism may be linked to degeneration of motor neurons [41]. Astrocytes play an important role in NVU function, interacting with and supporting neurons and blood vessels [42] and these changes in metabolic function in cortical astrocytes may contribute to NVU dysfunction in *db/db* mice.

Microglia are important modulators of homeostasis in the cerebral cortex. We found an increased number of microglia in the cortex of *db/db* mice, occurring in unison with overexpression of inflammatory pathways and genes involved in myelin damage, indicating neuronal injury in the diabetic brain [43]. ApoE is an established genetic risk factor for Alzheimer’s disease with roles in Aβ clearance, glucose metabolism and proinflammatory responses [44]. However, high levels of leptin can influence ApoE expression [45], therefore this finding should be explored in other diabetic models before a conclusion is made. In diabetic retinopathy, neurodegeneration is known to be an important early event (prior to vascular complications [46]), and is thought to be driven by chronic inflammation. A recent study showed that depleting microglia in a high-fat-diet-induced model of diabetes was able to improve cerebrovasculature deficits [47]. Further understanding of the neuroinflammatory pathways mediated by microglia in the type 2 diabetes cortex will be beneficial in understanding the role of neuroinflammation in the progress of diabetes-related cognitive decline.

Diabetes is known to have a profound effect on vascular function. Our IHC data indicated reduced laminin^+^ vascular density in the *db/db* mouse cortex, mirrored by increased acellular capillaries and loss of pericytes. This is in line with findings in the *db/db* retina, where vascular alterations can be seen from 10 weeks [48]. We also observed evidence of vascular leakage in specific ‘hot spots’ in the *db*/*db* mouse cortex, although this requires further characterisation. Top DEGs in endothelial cells suggested alterations to angiogenesis and vessel disease, while smooth muscle contraction and metabolism were dysregulated in pericytes. We saw a statistically significant reduction of ZO-1 protein expression in the parietal and temporal cortex of individuals with type 2 diabetes. We also observed region-specific increases in occludin expression, although the cellular source of this upregulation remains to be clarified. Loss of vascular density and pericyte coverage have been reported in diabetic retinopathy [49] and also in the cortex in Alzheimer’s disease [8], strengthening the assertion that progressive NVU dysfunction in the brain during type 2 diabetes is linked to neuronal depletion and cognitive decline. Together, these findings of altered neuronal maturation, astrocyte metabolism and microglial inflammation in the *db/db* mouse cortex highlight the impact of complex neuroglial interactions in type 2 diabetes-induced cognitive decline.

We report that *db/db* mouse cortex showed a significant increase in glycolysis and alterations to a number of genes involved in oxidative phosphorylation (e.g. *mt-Nd2* and *mt-Nd5* were reduced in immature neurons). This result suggests that at least some cells in the type 2 diabetes cortex may switch to anaerobic glycolysis as the main supply of energy (Warburg effect). Interestingly, this Warburg effect metabolism shift has been shown to drive neuronal degeneration in sporadic Alzheimer’s disease [50]. Metabolism was also dysregulated in mature neurons and astrocytes. Mitochondrial dysfunction in diabetes has been widely described [51]. We propose that altered metabolic function of key component cells of the brain NVU may play a major role in the loss of vascular integrity and homeostasis leading to increased risk of neurodegeneration and cognitive decline.

Our study reveals distinct changes to the cells of the cerebral cortex in type 2 diabetes. One limitation of our approach is the use of IHC quantification to validate changes to cortical NVU populations, as IHC is primarily a qualitative technique. Future analysis should focus on characterisation and quantitative validation of cortical cellular heterogeneity and subcellular vulnerability in type 2 diabetes. Our dataset facilitates opportunities for molecular analysis and their validation in cell–cell communication in diabetes-related cortical dysfunction. Future studies in such diabetes models will provide further insights into the therapeutic potential of targeting these gene alterations in the NVU for treatment of diabetic comorbidities of memory and cognition.

## Supplementary Information

ESM 1 (PDF 1.66 mb)

## Data Availability

The single-cell sequencing data that support this study are available at GEO accession GSE217665 (https://www.ncbi.nlm.nih.gov/geo/query/acc.cgi?acc=GSE217665)

## Abbreviations

DCX: Doublecortin
DEG: Differentially expressed gene
GFAP: Glial fibrillary acidic protein
GO: Gene ontology
IHC: Immunohistochemistry
MWM: Morris water maze
NeuN: Neuronal nuclear protein
NVU: Neurovascular unit
ROI: Region of interest
scRNA-seq: Single-cell RNA sequencing
SVZ: Subventricular zone
WGCN: Weighted gene correlation network
WT: Wild-type
