# Supplementary material for: Disruption of cortical cell type composition and function underlies diabetes-associated cognitive decline
Source: Diabetologia. 2023 Jun 23;66(8):1557–75. doi: 10.1007/s00125-023-05935-2 (PMC10317904; doi:10.1007/s00125-023-05935-2)
Supplement: Supplementary file 1 — (PDF 1.66 mb) [file 125_2023_5935_MOESM1_ESM.pdf]

Electronic Supplementary Material

Methods:

Animals

Body weight, postprandial blood glucose and insulin levels were determined immediately before sacrifice in db/db and control mice. Blood glucose levels were measured from nicked tail using the glucometer Optium Xceed (Abbott, UK). Blood samples were centrifuged during 7 min, 6500 rpm at 4°C, and plasma fraction was stored at −80 °C until used. Plasma insulin levels were measured using an ultrasensitive mouse enzyme-linked immunosorbent assay (10-1251-01, Mercodia Inc., Winston-Salem, NC, USA).

Actimetry and new object discrimination (NOD) task

Eight days prior to sacrifice spontaneous locomotor activity was assessed by measuring the walking velocity for 30 minutes in a rectangular box (44 cm long x 22 cm width x 40 cm high) the day before starting to analyse episodic memory with the NOD test. Whilst episodic memory is largely associated with the entorhinal cortex, there is also evidence that other cortical brain regions may be involved in episodic memory such as the inferior temporal cortex, the hippocampus, the fornix, the mammillary bodies, the mediodorsal nucleus of the thalamus, and regions of the prefrontal cortex.

On day 1 animals were exposed to two objects, for habituation purposes not used again. On day 2 mice received two sample trials and a test trial. Firstly, mice were exposed to 4 copies of a novel object (blue balls) arranged in a triangle-shaped spatial configuration and allowed to explore them for 5 min. After 30 min, the mice received a second sample trial with 4 novel objects (red cones) in a quadratic-shaped spatial configuration, for 5 min. After a delay of 30 min, the mice received a test trial with 2 copies of the object from sample trial 2 (“recent” objects) placed at the same position, and two copies of the object from sample trial 1 (“familiar”

objects) placed one of them at the same position ("old non displaced" object) and the another in a new position ("familiar displaced" object). Discrimination index was calculated as the percentage of time exploring recent objects/total exploration time.

Morris Water Maze

Experiments commenced the day after the conclusion of the NOD test. The maze consisted of a round tank of water (90 cm in diameter) with four equal virtual quadrants indicated with geometric cues mounted on the walls. Water temperature was 21±2ºC. The escape platform was located 2-3 cm below the surface and hidden by coloring the water with kaolinite (0102, Biocop, Barcelona, Spain). The acquisition phase was run for 4 days consisted of 8 sessions (2 sessions per day) of 2 trials each with the platform submerged in quadrant 2. The time limit was 60 s per trial with a 10 min intertrial break. When the animal did not find the platform, it was placed on it for 10 s. The retention phase started a day after acquisition phase was completed and consisted in a single trial with the platform removed.

scRNA-seq library preparation, sequencing, and associated analysis

Raw sequencing reads were aligned to the pre-mRNA annotated Mus musculus reference genome version GRCm38 and counts were estimated using Cellranger (10x Genomics). The generated cell-by-gene unique molecular identifier (UMI) count matrix was analysed using Seurat. Only cells expressing at least 250 genes, 500 transcripts and genes with expression in at least 3 cells were kept. The cells were also filtered by the maximum of 20% mitochondrial genes. The UMI counts were then normalized for each cell by the total expression, multiplied by 10,000 and log transformed. Seurat's default method to identify highly variable genes and scale data for regressing out variation from UMI and mitochondrial genes was used. The scaled data with variable genes were used to perform principal component analysis.

Identification of Cell-types, marker-genes, and differential expression analysis

Genes were projected into principal component (PC) space using the principal component analysis (RunPCA). The top 15 principal components were chosen for further analysis, including clustering to identify cell populations. First 15 PCs were used as inputs into Seurat's FindNeighbors, FindClusters (at 0.8 resolution) and RunUMAP functions. In brief, a shared-nearest-neighbour graph was constructed based on the Euclidean distance metric in PC space, and cells were clustered using the Louvain method. RunUMAP functions with default settings were used to calculate two-dimensional UMAP coordinates and search for distinct cell populations. Positive differential expression of each cluster against all other clusters (model-based analysis of single-cell transcriptomics; MAST) was used to identify marker genes for each cluster. Markers used for identification are shown in the main text Table 2.

Differential gene expression of samples or comparing cell type subclusters was done using

the MAST algorithm, which implements a two-part hurdle model. Seurat natural log (fold change) > 0.25 (absolute value), adjusted P value (Bonferroni correction) < 0.05, and expression in greater than 10% of cells in both comparison groups were required to consider a gene differentially expressed for cluster analysis. Further, more stringent values were used for gene selection. Biological pathway and gene ontology enrichment analyses were performed using Enrichr with the input species set Mus musculus.

Single cell regulatory network for db/+ and db/db cell populations was performed using pySCENIC platform. Specifically, pySCENIC 0.12.0 and ctxcore 0.2.0 was used. GRNBoost2 (https://github.com/tmoerman/arboreto) in pySCENIC was applied to infer gene regulatory networks from count matrix file. Then potential direct-binding targets (regulons) were selected based on DNA-motif analysis. Finally, gene regulatory network activity for individual cells was identified. To find the regulators for each cell type, the regulon specificity score (rss) was calculated, and regulons were ranked accordingly.

Weighted gene correlation analysis

A weighted gene co-expression network (WGCN) was created by the WGCNA R package to reveal correlations between cell type gene expression. To construct the WGCN with scale-free topology, different values of soft thresholding power β were assessed for the network topology analysis, and the value of 9 was selected. The Pearson correlation coefficient and the signed network options were used to measure the correlation between the expression of each pair of genes and to maintain only positive correlations, respectively. Modules in the WGCN were depicted in different colors and the relationships between the modules were depicted by module eigengenes that are the first principal component of the expressions in modules. These principal genes were used for Gene ontology and biological pathway analysis.

Pseudotime trajectory analysis

R based programme Monocle was used to order cortical cells in a pseudotime trajectory. Monocle learns a trajectory that reconstructs the progress of a cell in a cell differentiation process. Cells were clustered in Seurat, then neuronal and glial clusters were selected as input into Monocle to infer the transcriptional profile progression between the control and db/db cortex. Specifically, UMAP embeddings and cell subclusters generated from Seurat were converted to a cell_data_set object using SeuratWrappers (v.0.2.0) and then used as input to perform trajectory graph learning and pseudotime measurement through independent component analysis (ICA) with Monocle.

Cresyl Violet Staining

Briefly, after dehydration in 70% ethanol for 15 minutes, sections were incubated in cresyl violet (Sigma, St. Louis, MO, USA) solution 0.5% w/v for 10 minutes. After washing, sections were fixed in 0.25% acetic acid in ethanol for 5 minutes and subsequent 100% ethanol and xylene for 2 minutes. Sections were mounted with DPX (317616, Sigma, St. Louis, MO, USA).

Metabolic assessment of cortical brain punches

Briefly, acute coronal brain slices (250 µM) were cut from db/db and db/+ mice using the Leica VT100S vibratome immediately after sacrifice by cervical dislocation. Slices were maintained in ice cold artificial cerebrospinal fluid (aCSF) containing 10mM sucrose, and 1mm punches were taken from the cortex. 7-8 punches per group were transferred directly to a 96 well plate (Agilent) containing 180 µL ice-cold a-CSF-sucrose, ensuring the punch was centred in the well. The plate was left to rest in a 37oC incubator for 1 hour before metabolic profiling was carried out in the Seahorse XFE96 (Agilent).

Patient Tissue

The tissues were snap-frozen and ten-µm thick sections of the tissue specimens were cut at -20°C using a Microm Cryo Star HM 560 cryostat (Thermo Fisher Scientific) and stored at -80°C until further use. Cryostat sections were air dried at room temperature for 20 min before immunofluorescence staining. The sections were fixed in 4% (w/v) formaldehyde (28908, Thermo Fisher Scientific) for 20 min and were washed once in 3x PBS. Individual sections were encircled with a hydrophobic barrier using a PAP pen (ADI-950-233-0001, Biocompare). The sections were treated for 1h with 1x PBS (70011044, Gibco) supplemented with 10% normal goat serum (006-01, Southern Biotech) and 0.1% Triton X-100 (T8787, Sigma). Lipofuscin autofluorescence was reduced by treating the sections with Trueblack (23007, Biotium) diluted 20x in 70% ethanol. The slides were washed three times with PBS. Next, the sections were incubated overnight with primary antibodies at 4°C as indicated in Table 3. Antibodies were diluted in Normal Antibody Diluent (phosphate buffered- lab. AB999, Scytek). Sections were subsequently washed three times with PBS and incubated with both secondary antibodies (1) goat anti-mouse Alexa Fluor™ 488 and goat anti-rabbit Alexa Fluor™ 633 or (2) goat anti-rabbit Alexa Fluor™ 488 and goat anti-mouse Alexa Fluor™ 633.

After 1 h incubation at room temperature, the slides were washed three times with PBS. For wheat germ agglutin (WGA), incubation was performed after secondary antibody incubation. Sections were incubated with WGA-AF555 for 30 min at room temperature. After incubation with WGA, the slides were again washed three times with PBS. After the final washes, slides were mounted with Vectashield antifade mounting medium with DAPI (H-1200-10, Vector Laboratories) to preserve fluorescence.

Western blotting of patient samples

Brain proteins were extracted in RIPA lysis buffer (50 mM Tris pH 8, 150 mM NaCl, 1% IGEPAL CA-630, 0.5% sodium deoxycholate, 0.1% sodium dodecyl sulfate) containing 1x complete EDTA-free protease inhibitor cocktail (Roche, Germany) and maintained on ice. They were sonicated until there were no visible aggregates. Protein concentration was determined by the Modified Lowry kit (DC protein assay, Bio-Rad, USA). 50 to 150 µg of protein were resolved in SDS-PAGE, and transferred onto nitrocellulose membranes (Millipore, Spain). Membranes were then blocked in Tris-buffered saline containing 1% Tween-20 (TBS-T) and 10% non-fat dry milk for 1h at room temperature. After two washes in TBS-T, membranes were incubated overnight at 4ºC under constant agitation with corresponding primary antibodies at the appropriate dilutions (GFAP, Iba-1, NeuN, Occludin, PDGFRα/β, PSD95 and ZO-1) in TBS-T with 5% non-fat dry milk. The next day, after 3 washes in TBS-T, membranes were incubated with corresponding peroxidase-conjugated secondary antibodies diluted in blocking solution (Goat anti-Rabbit IgG, Sigma-Aldrich, Cat# A0545, 1:5,000; Rabbit anti-Mouse IgG, Sigma-Aldrich, Cat# A9044, 1:5,000, Rabbit anti-Goat IgG, Sigma-Aldrich, Cat# A5420, 1:5,000) for 1h at room temperature under agitation. After 3 washes in TBS-T, signal was developed using the EZ-ECL Enhanced Chemiluminescence Detection Kit for HRP (Thermo-Invitrogen, USA) and captured with Fuji X-ray films Super RX-N (Fujifilm, Japan). Band intensities were quantified using Image J

software, version 1.53a. Proteins were normalized using Ponceau staining of the membranes, to ensure same load in each lane.

ESM Table 1. Characteristics of control and type-2 diabetic donors included in western blot study

Sex	Age (Years)	PMD (hours)	T2D	AD Braak Stage	Comorbidities
FEMALE	78	24	NO	I-II	-
FEMALE	77	20	NO	I-II	-
FEMALE	68	13	NO	I-II	-
Mean	*74.3 ± 5.5*	*16.5 ± 4.9*			
MALE	78	11	YES	Not Assessed	Vascular encephalopathy
FEMALE	95	20	YES	II	Vascular encephalopathy, Arterial hypertension, Chronic Obstructive Pulmonary Disease
FEMALE	88	24	YES	II	AgD II, Cerebral Amyloid Angiopathy, Arterial hypertension, Small Vessel Disease, Ovarian Cancer Surgery
Mean	*87.0 ± 8.5*	*18.3 ± 6.6*			

PMD: Post-mortem Delay, AgD: Argyrophilic grain disease

ESM Table 2. Swimming and walking speed in db/db and control mice.

	Swimming speed (cm/s)	Walking speed (cm/s)
Control	11.89 ±0.28	4.29±0.23
db/db	4.69 ±0.38**	3.68 ±0.23

Swimming speed was significantly compromised in db/db animals (**p<0.001 vs. control (WT)) probably due to their tendency to float. No differences in walking speed were observed in the actimetry test (p=0.104) suggesting that motor activity is not severely compromised in these animals.

Supplementary figures

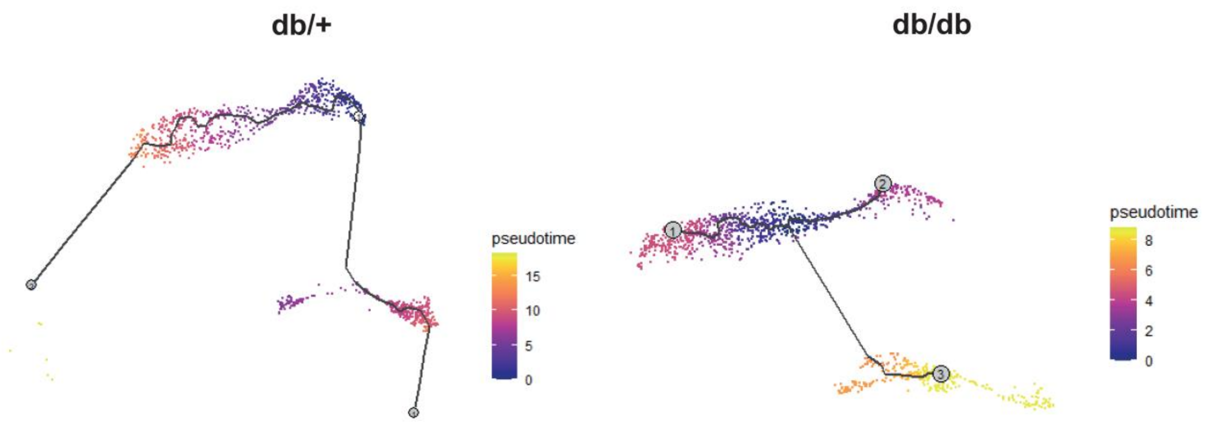

ESM Figure 1. Pseudotime trajectory on glia populations (astrocytes and oligodendrocytes together). Control (db/+) and db/db indicating the cellular heterogeneity.

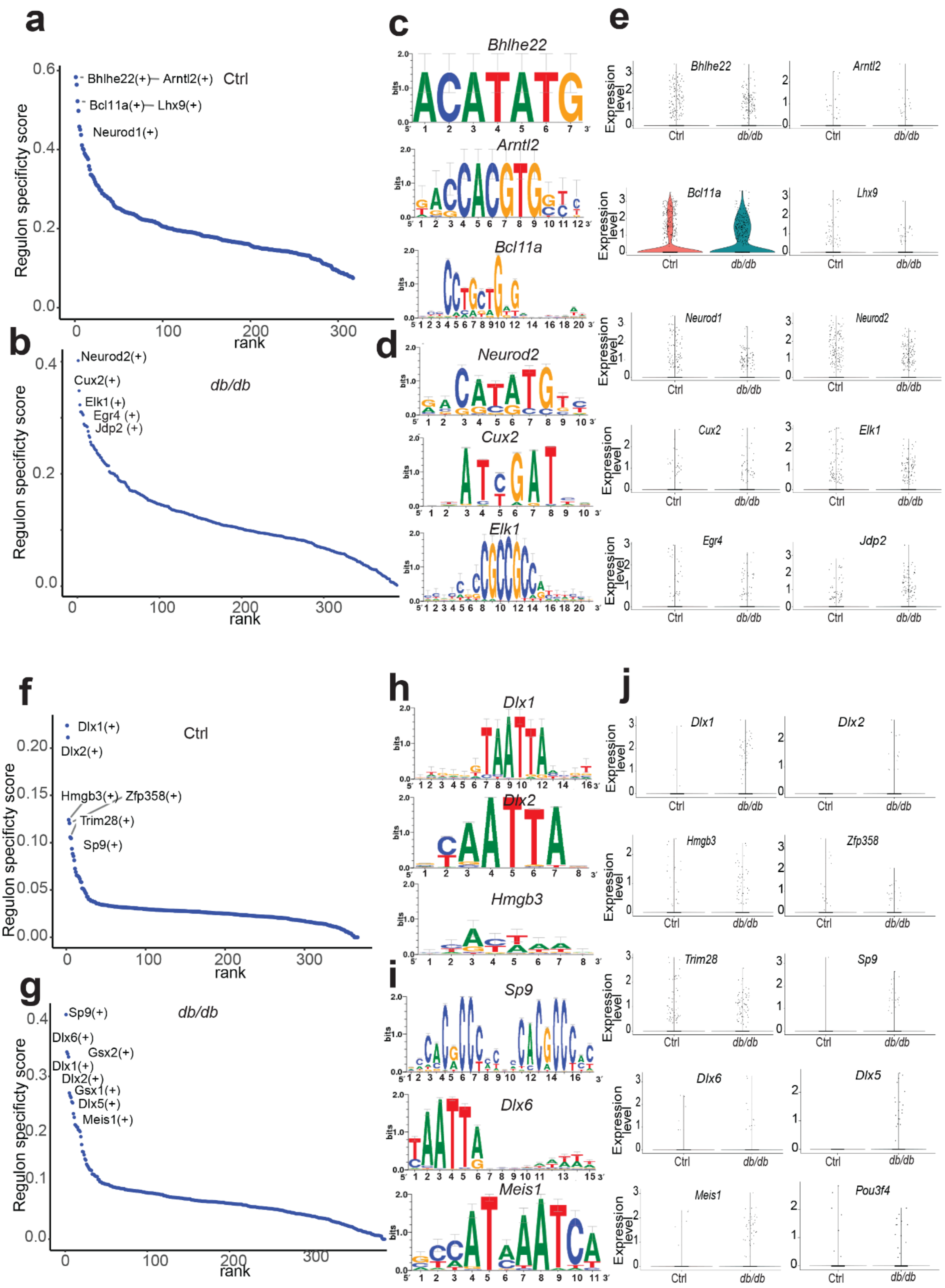

162

163 **ESM Figure 2. Single-Cell Regulatory Network Inference and Clustering Analysis.** (a–d)

164 Top regulons (a, b) and top unique motifs (c, d) in db/+ (a, c) and db/db (b, d) mouse mature

neurons. (e) Differential expression patterns of top regulon genes in db/+ and db/db mouse mature neuronal cell populations. (f–j) Top regulons (f, g) and unique motifs (h, i) in db/+ (f, h) and db/db (g, i) mouse immature neurons. (j) Differential expression patterns of top regulon genes in db/+ and db/db mouse immature neuronal cell populations. Expression levels for individual genes are presented as the normalised counts.

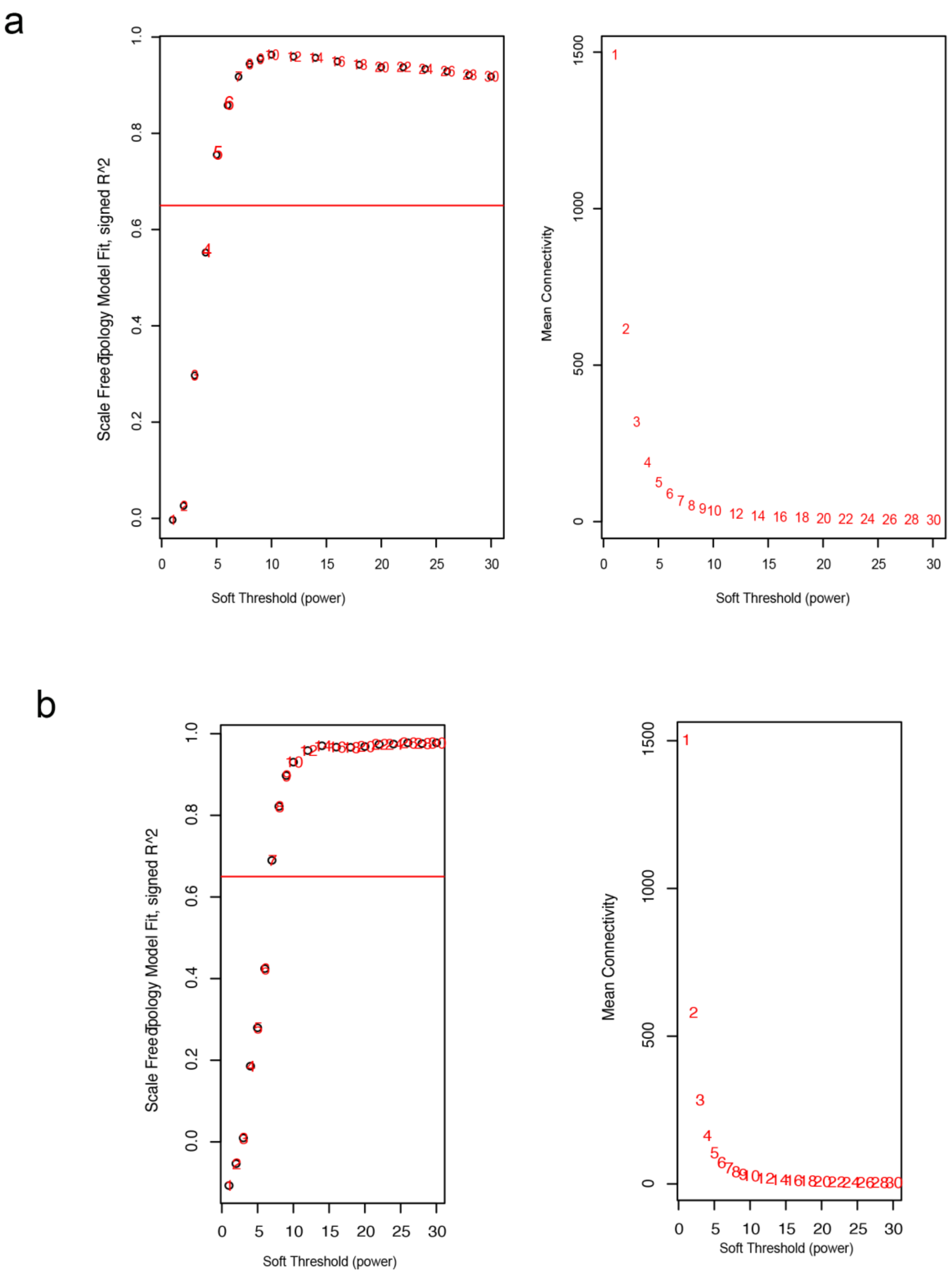

ESM Figure 3: Scale independence and mean connectivity of correlated gene modules. (a) Control cortex and **(b)** *db/db* cortex.

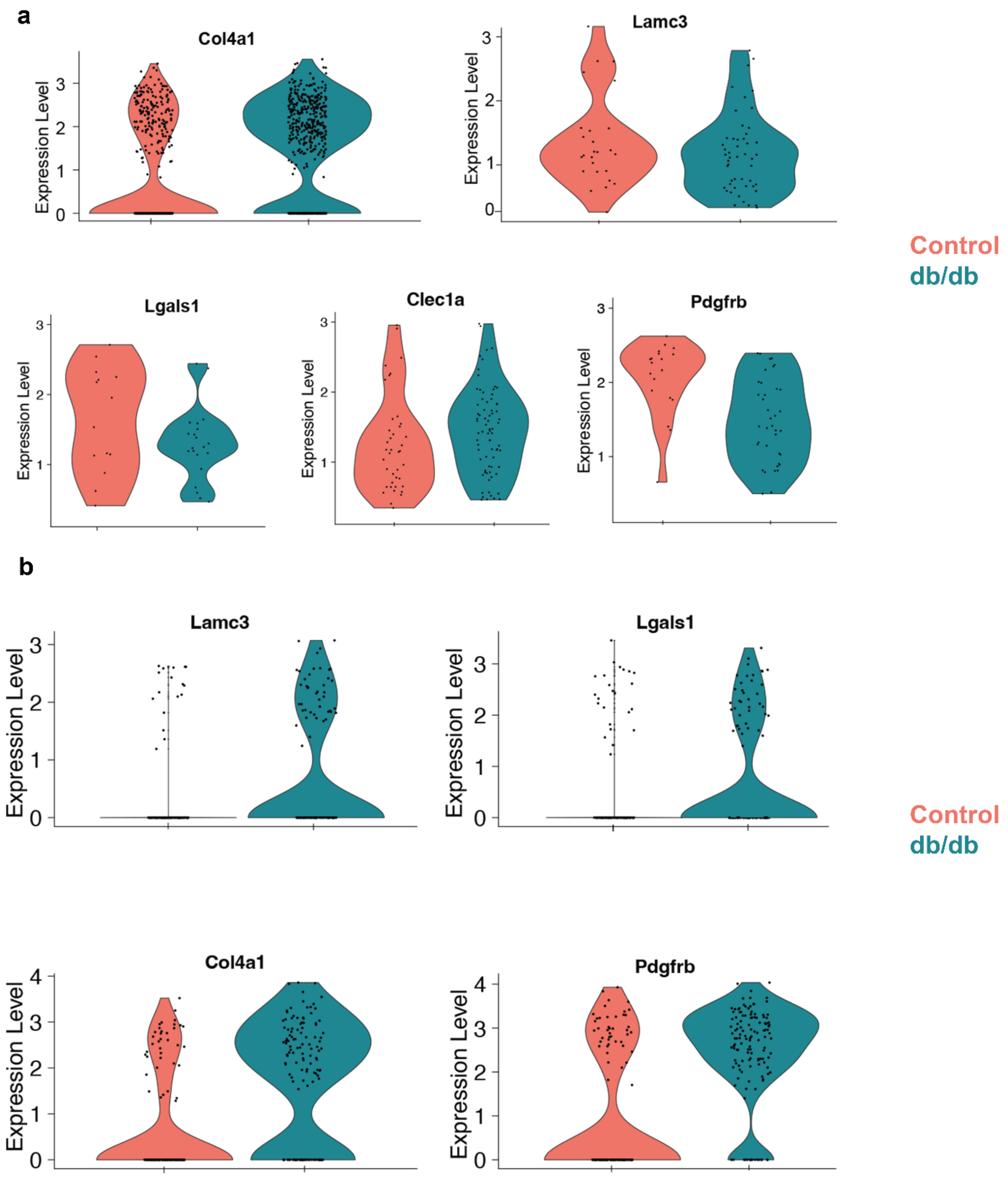

ESM Figure 4: Marker gene expression in endothelial cells and pericytes (a) Marker gene expression in endothelial cells in control (red) and *db/db* (blue) cortex. **(b)** Marker gene expression in pericytes in control (red) and *db/db* (blue) cortex.

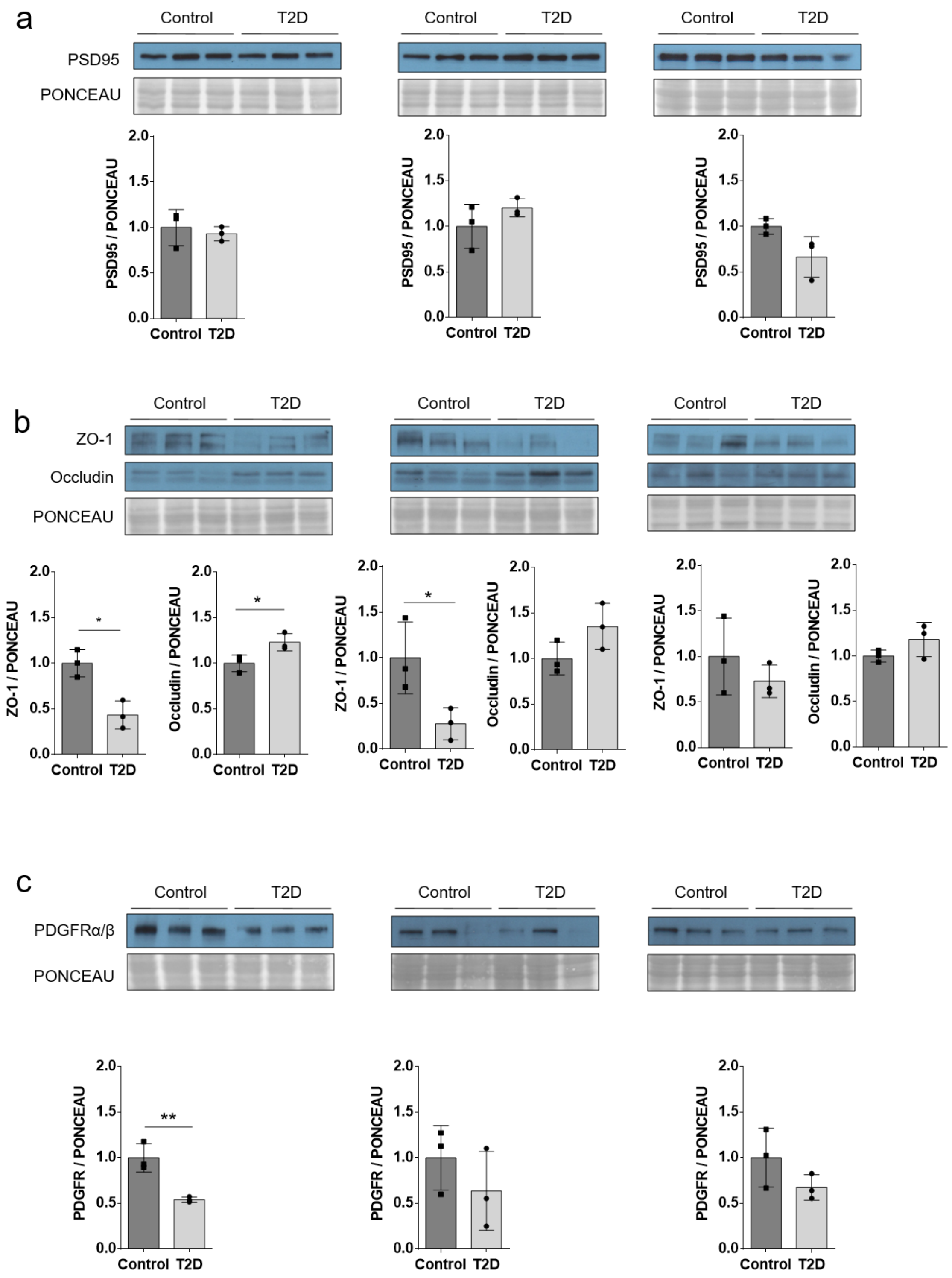

ESM Figure 5: Further analysis of Cortical NVU markers in T2D patients. Western blotting of human cortex samples (Parietal, Temporal and Frontal) shows the expression of (a) PSD95, (b) ZO-1 and occludin and (c) PDGFR α/β. N=3, * $p<0.05$ vs. Control group.
